# Challenges and insights in the exploration of the low abundance human ocular surface microbiome

**DOI:** 10.3389/fcimb.2023.1232147

**Published:** 2023-09-01

**Authors:** Elio L. Herzog, Marco Kreuzer, Martin S. Zinkernagel, Denise C. Zysset-Burri

**Affiliations:** ^1^ Department of Ophthalmology, Inselspital, Bern University Hospital, University of Bern, Bern, Switzerland; ^2^ Department for BioMedical Research, University of Bern, Bern, Switzerland; ^3^ Graduate School for Cellular and Biomedical Sciences, University of Bern, Bern, Switzerland; ^4^ Interfaculty Bioinformatics Unit and Swiss Institute of Bioinformatics, University of Bern, Bern, Switzerland

**Keywords:** low abundance microbiome, ocular surface microbiome, whole metagenome shotgun sequencing, DNA extraction kit, nylon flocked swab, host DNA depletion, mechanical lysis, taxonomic profiling

## Abstract

**Purpose:**

The low microbial abundance on the ocular surface results in challenges in the characterization of its microbiome. The purpose of this study was to reveal factors introducing bias in the pipeline from sample collection to data analysis of low-abundant microbiomes.

**Methods:**

Lower conjunctiva and lower lid swabs were collected from six participants using either standard cotton or flocked nylon swabs. Microbial DNA was isolated with two different kits (with or without prior host DNA depletion and mechanical lysis), followed by whole-metagenome shotgun sequencing with a high sequencing depth set at 60 million reads per sample. The relative microbial compositions were generated using the two different tools MetaPhlan3 and Kraken2.

**Results:**

The total amount of extracted DNA was increased by using nylon flocked swabs on the lower conjunctiva. In total, 269 microbial species were detected. The most abundant bacterial phyla were *Actinobacteria*, *Firmicutes* and *Proteobacteria*. Depending on the DNA extraction kit and tool used for profiling, the microbial composition and the relative abundance of viruses varied.

**Conclusion:**

The microbial composition on the ocular surface is not dependent on the swab type, but on the DNA extraction method and profiling tool. These factors have to be considered in further studies about the ocular surface microbiome and other sparsely colonized microbiomes in order to improve data reproducibility. Understanding challenges and biases in the characterization of the ocular surface microbiome may set the basis for microbiome-altering interventions for treatment of ocular surface associated diseases.

## Introduction

The human ocular surface has long thought to be sterile due to the presence of antimicrobial components in the tear film (McDermott, 2013) and continuous motion of the eye lids (Shovlin et al., 2013). First investigations of conjunctiva swab cultures 92 years ago described 43% of samples as absolutely sterile. The predominating genus in the remaining cultures was *Staphylococcus* (Keilty, 1930). The idea that the ocular surface is sterile or only periodically colonized during infections has been changed with the introduction of modern sequencing technologies such as 16S rRNA gene sequencing and whole-metagenome shotgun sequencing (Doan et al., 2016). A resident microbiome on the ocular surface, as found in other mucosal sites throughout the body, has been described recently (Human Microbiome Project C, 2012a; Human Microbiome Project C, 2012b). Previous studies using either 16S rRNA gene sequencing or whole-metagenome shotgun sequencing, have shown that the three dominant microbial phyla of the ocular surface microbiome (OSM) are *Proteobacteria*, *Firmicutes* and *Actinobacteria* (Dong et al., 2011; Zysset-Burri et al., 2021) with highly variable abundances between individuals. To our current knowledge, the taxonomic composition of the OSM is influenced by environmental and demographic factors such as age (Zhou et al., 2014; Cavuoto et al., 2018b; Cavuoto et al., 2018a) but not by sex (Zhou et al., 2014; Ozkan et al., 2017; Cavuoto et al., 2018b; Cavuoto et al., 2018a; Suzuki et al., 2020). To date, the impact of DNA extraction kits on OSM data has not been explored in respect to microbial composition using whole-metagenome shotgun sequencing. Previous studies investigated the effects of swabbing technique (soft versus deep) (Dong et al., 2011), topical anesthetics (Delbeke et al., 2022) and temporal stability (Ozkan et al., 2017) on the outcome of OSM taxonomic profiling. It has been postulated that even the intraocular environment has its own microbiome in healthy and diseased eyes (Deng et al., 2021). Moreover, it has been suggested that, in addition to the tear film, eye lid motion and antimicrobial peptides, the OSM itself contributes to ocular surface health. The OSM’s contribution is mediated by selective immune tolerance to commensal specific compounds (Ueta and Kinoshita, 2010) and colonization resistance (St Leger et al., 2017), showing the importance of the OSM in maintaining a healthy ocular surface and preventing infectious and inflammatory diseases.

Low microbial biomass combined with low levels of microbial DNA on the ocular surface lead to similar challenges as in the characterization of blood or placental microbiomes, with contamination issues outcompeting the biological signal (Glassing et al., 2016; Lauder et al., 2016). A bacterial density of 0.06 bacteria per human cell was measured by broad range 16S rDNA gene qPCR in conjunctival swab samples, around 200 times less than on the buccal mucosa (12 bacteria per human cell) and > 250 times less than on facial skin (16 bacteria per human cell) (Doan et al., 2016). Even with this low density in biomass, modern sequencing techniques are able to determine microbial compositions, but suffer from contamination problems with decreasing microbial concentration. This was shown by Brandt and Albertsen 2018, where pure water spiked with *Escherichia coli* in varying concentration was analyzed by 16S rRNA sequencing and samples with 10 bacterial cells/ml showed 8% contaminating sequences. To circumvent these issues, we tested the following settings: sample collection using two different swab types (standard cotton versus flocked nylon swabs), isolation of microbial DNA by two different DNA extraction kits, quantification of bacterial 16S rRNA gene DNA with qPCR, an increased sequencing depth compared to earlier studies and inclusion of positive and no template controls (NTC) for each individual step of the pipeline. The two DNA extraction kits were chosen due to their use in previous studies (Omega (Zysset-Burri et al., 2021),) and their availability, combination of mechanical and enzymatic lysis, enzymatic host DNA depletion and low input material (Qiagen). While enzymatic host DNA depletion may not be the most efficient method to deplete host DNA compared to paramagnetic beads and DNA methylation traps (Ganda et al., 2021), a more than three- to then-fold increase in relative bacterial DNA was observed (Marotz et al., 2018; Heravi et al., 2020). The main goal of this study was to compare the impact of different sampling and analysis methods on the overall structure of OSM, while the described species itself may not be of clinical relevance.

## Methods

### Recruitment and study design

This study was approved by the Ethics Committee of the Canton of Bern (ClinicalTrials.gov: NCT04658238). The procedures followed the principles of the Declaration of Helsinki and the International Ethical Guidelines for Biomedical Research involving Human Subjects. Each study subject was informed about the procedures and purpose of the study and signed informed consent before study enrollment. All study subjects were enrolled at the Department of Ophthalmology of the Inselspital, University Hospital Bern in Bern, Switzerland. Inclusion criteria consisted of willingness to sign an informed consent and an age of 18 years or older. Subjects were excluded if they were not willing to or able to sign an informed consent, were younger than 18 years of age, had received systemic or topical antibiotics in the last three months, were using medical eye drops or underwent ocular surgery within the last three months. The recruited study cohort consists of six females with a mean age of 48 years (SD = 11.46) at sampling.

### Sample collection

**Figure 1:**
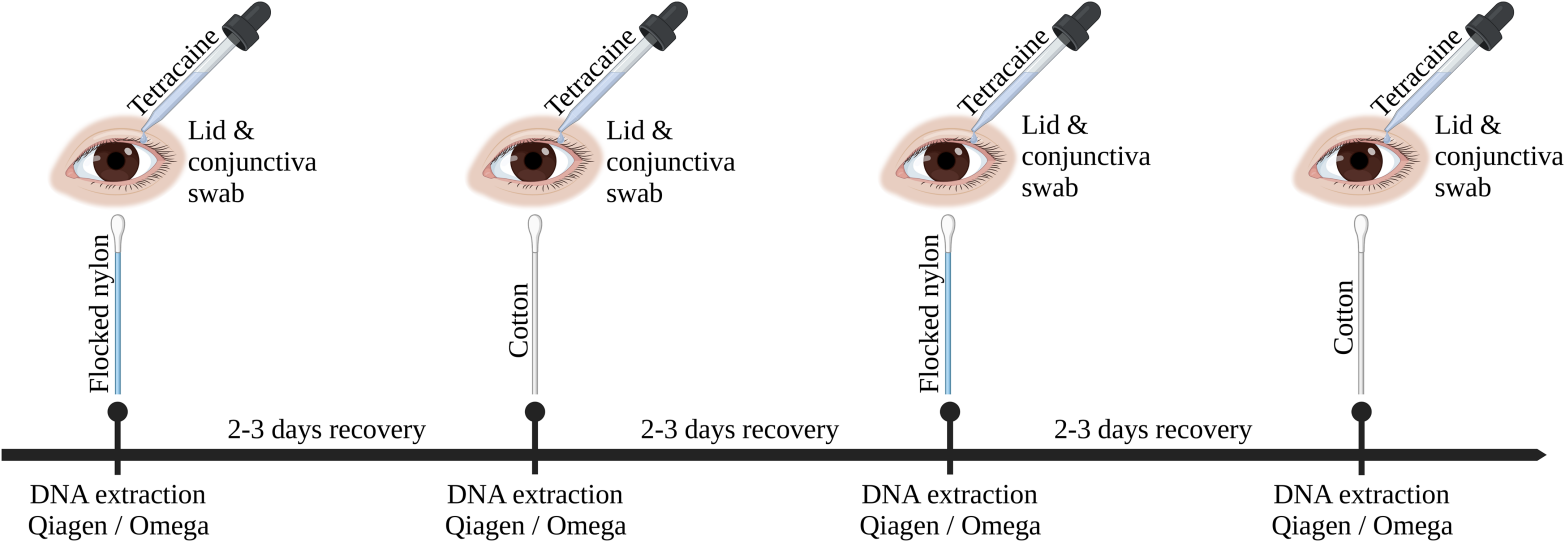
Graphical overview of sampling procedure. This timeline was repeated three times with each DNA extraction kit (Omega, Qiagen without host DNA depletion, Qiagen). Ocular surface swabs were alternating taken using flocked nylon swabs or cotton swabs. Ocular swabbing was performed under local anesthesia (Tetracaine 1%).

Ocular swab samples were collected from six subjects with healthy eyes at four time points over the course of two weeks for each DNA extraction kit. The first and third sampling was performed with flocked nylon swabs (FLOQSwabs #518CS01, Copan, Brescia, Italy), the second and fourth sampling with standard cotton swabs (Catalogue #1501256, Applimed SA, Châtel-St-Denis, Switzerland). A gap of two to three days were kept between each sampling for recovery reasons (**Figure 1**). The ocular surfaces of patients were anesthetized with one drop of Tetracaine 1% solution per eye (Tetracaine 1% SDU Faure, Théa, Clermont-Ferrand, France). Conjunctiva swabs were taken by swabbing over the lower conjunctiva three times counter-rotating the swab to the direction of movement. After conjunctiva swab collection, the Meibomian glands of the lower eye lid were expressed from caudal to cranial using a sterile cotton swab before the lid swab collection. Lower lid swabs were collected by swabbing three times over the lower lid, counter-rotating the swab to the direction of movement to increase the contact area between swab head and eye lid. Experimental swabs were stored in either a dry microcentrifuge tube on ice or in one milliliter of ice cooled DPBS 1X (#14190185, Fisher Scientific Gibco, Reinach, Switzerland), according to the protocols used for DNA extraction. NTCs were produced by processing empty swabs and swabs that absorbed one drop of 1% Tetracaine 1% with the same protocol as the corresponding extraction kit. Swabs were kept on ice for no longer than two hours before DNA extraction.

### DNA extraction

Whole DNA was extracted with two different kits, the QIAamp DNA Microbiome Kit (51704) from QIAGEN (Hilden, Germany) and the E.Z.N.A. MicroElute Genomic DNA Kit (D3096-02) from Omega Bio-Tek (Norcross, USA). These two kits are further referred to as Qiagen or Omega, respectively.

Omega and Qiagen DNA extractions were performed according to the provided protocol for swabs with minor changes (**Supplementary material S1**). The Qiagen extraction kits were further split into Qiagen1 and Qiagen2 in data analysis, representing two different batches of kits with two different lot numbers. In extractions with the Qiagen1 kit, the enzymatic host DNA depletion did not work as well as intended. Therefore, this sampling was repeated with the same subjects and same sampling methods approximately four months after the initial Qiagen1 sampling and seven months after the Omega sampling, receiving the name Qiagen2.

DNA extraction eluates were measured on a spectrophotometer (NanoDrop 1000, Thermo Scientific, Waltham, Massachusetts, USA) to assess solvent and salt contaminations prior to storage at -20°C.

DNA from 18 aliquots of a positive control with known bacterial composition (ZymoBIOMICS Microbial Community Standard, D6300, Irvine, CA, USA) were extracted using both DNA extraction kits with the same protocol used in OSM DNA extractions. Additionally, eluates from these microbial community standard DNA extractions were diluted to the same DNA concentration found in OSM samples. Dilutions were made with the elution buffer of the Omega or Qiagen kit, respectively. This provided information about the accuracy of the sequencing pipeline at low DNA concentrations.

### Quantification of bacterial DNA

Bacterial DNA content was assessed using qPCR of the 16S rRNA gene. The primers used in qPCR were taken from a publication by Galazzo et al., 2020 (primer pair 16S-341_F CCTACGGGNGGCWGCAG and 16S-805_R GACTACHVGGGTATCTAATCC) (Galazzo et al., 2020). As a master mix iTaq Universal SYBR Green Supermix (Bio Rad, Hercules, CA, USA) was used in a 10 μl reaction with a primer concentration of 300 nM. All samples were measured in triplicate and run on a CFX Connect Real-Time System (Bio Rad, Hercules, CA, USA). The PCR amplification program was initialized by a 3 min at 95°C denaturation followed by 40 two-step amplifications set at 95°C for 5 s and 60°C for 30 s. Melting curves were retrieved at the end of the amplification cycles and used to confirm amplification of the desired product. NTCs as well as a positive control stool sample with a high concentration of DNA were included in each plate. Cycle numbers were normalized to the expression of the higher concentrated positive stool sample control.

### Library preparation and metagenomic DNA sequencing

DNA concentration and quality were assessed prior to sequencing using a fluorometer (QubitFlex Fluorometer, Qubit 1X dsDNA HS Assay Kit #Q33231, Thermofisher Waltham, Massachusetts, USA). Due to the low DNA concentrations, the sequencing libraries in all samples were amplified with 12 PCR cycles. In samples with a library concentration below 1 nM after the initial PCR amplification (20.18% in total, 2.38% in Omega, 31.88% in Qiagen) the amplification was repeated with a total of 18 PCR cycles. If the libraries still not met the requirements, the samples were excluded from further analysis (7.02%). Libraries were prepared and sequenced by the Next Generation Sequencing Platform of the University of Bern, Switzerland. Sequencing libraries were prepared using the Illumina DNA Prep, (M) Tagmentation kit (#20018705, Illumina, San Diego, CA, USA), with four index sets (IDT for Illumina DNA/RNA UD Indexes Set A, B, C and D, #20027312, #20027214, #20042666, #20042667, Illumina). Samples were sequenced by Illumina NovaSeq 6000 on S4 flow cells. Paired end reads of 150 bp length were selected for this project and a sequencing depth of 60 million reads was aimed for.

### Annotation of sequencing reads

The raw reads were trimmed using trimmomatic (Version 0.36) with the options ‘LEADING:3 TRAILING:3 SLIDINGWINDOW:4:20 MINLEN:30’ (Bolger et al., 2014). Next, the quality filtered reads were mapped to the human reference genome (Ensembl GRCh38) using bowtie2 (Version 2.3.4.1) with default options (Langmead and Salzberg, 2012). The resulting SAM file was converted to a BAM file with samtools view (Version 1.10) and sorted with samtools sort (Danecek et al., 2021). The unmapped reads were extracted using with samtools bam2fq. The quality of the resulting filtered reads were checked with fastQC (Version 0.11.7) (Andrews, 2010).

Taxonomic annotation of sequencing reads was performed using the Metagenomic Phylogenetic Analysis tool (MetaPhlAn 3, version 3.0.14) with ChocoPhlAn 3 (version mpa_v30_CHOCOPhlAn_201901) as reference pangenome database (Beghini et al., 2021). Alternatively, Kraken2 (version kraken2_2.0.9beta) with the relative abundance estimation tool Bracken (version bracken_2.6.0) was used to observe the effect of different taxonomic annotation methods (Lu et al., 2017; Wood et al., 2019). The relative abundances of the annotated reads were calculated to determine the taxonomic composition of the OSM.

### Statistical analysis

Graphical representation of sequencing data and statistical analyses were produced using R (Version 4.2.1) and the R package ggplot2 (Version 3.3.6). The R package MaAsLin2 (Version 1.10.0) was used to create a mixed effect model with the DNA extraction method, swab type and sequencing run as fixed effects and with the study ID and age as random effects. Log transformation and normalization of the data were disabled. Subject IDs were set as random effect in MaAsLin2 to account for temporal dependence.

Statistical comparisons were performed with the following tests: Pairwise Wilcoxon rank sum tests, Students *t*-tests, One-way ANOVAs or PERMANOVAs (R package vegan (Version 2.6.2)). PCA and MaAsLin2 analyses are based on relative taxonomic abundance tables on species level. PCA analysis was performed with the R package vegan (Version 2.6.2).

## Results

### Amount of extracted DNA

**Figure 2:**
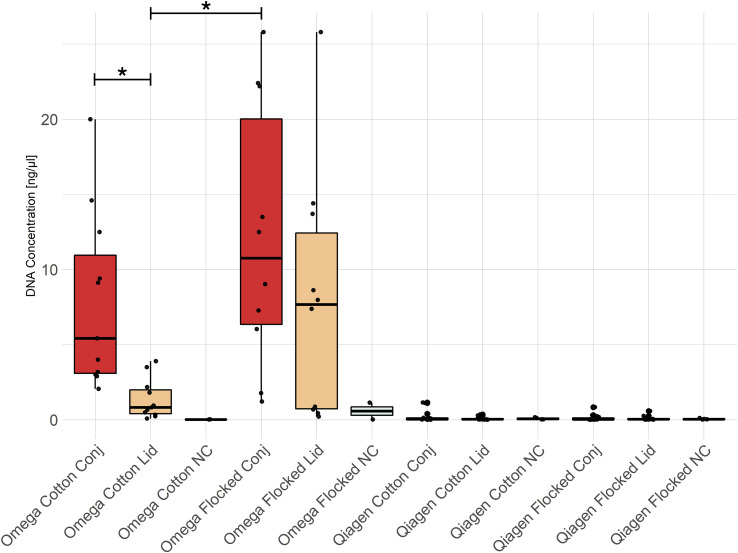
DNA concentration of swab extractions. All Omega samples, except the negative controls, showed significantly higher DNA concentrations compared to any group extracted with the Qiagen kit. Conj, conjunctiva; NC, No-template control. * = p-value < 0.05.

The total amount of extracted DNA in ocular swabs differed depending on the swab type, sampling location and DNA extraction kit (**Figure 2**). Independent of location and swab type, less DNA was extracted by the Qiagen compared to the Omega kit. The samples isolated by the Qiagen kit did not differ in DNA concentration from negative extraction controls according to Qubit fluorometer measurements. In the Omega kit, conjunctiva swab samples contained more DNA compared to lid swab samples using cotton swabs (Wilcoxon rank sum test, p-value = 0.001). Additionally, there was an increase in the amount of DNA in conjunctiva samples sampled with nylon flocked swabs compared to lid samples sampled with cotton swabs (Wilcoxon rank sum test, p-value = 0.001).

### Quantification of bacterial DNA

**Figure 3:**
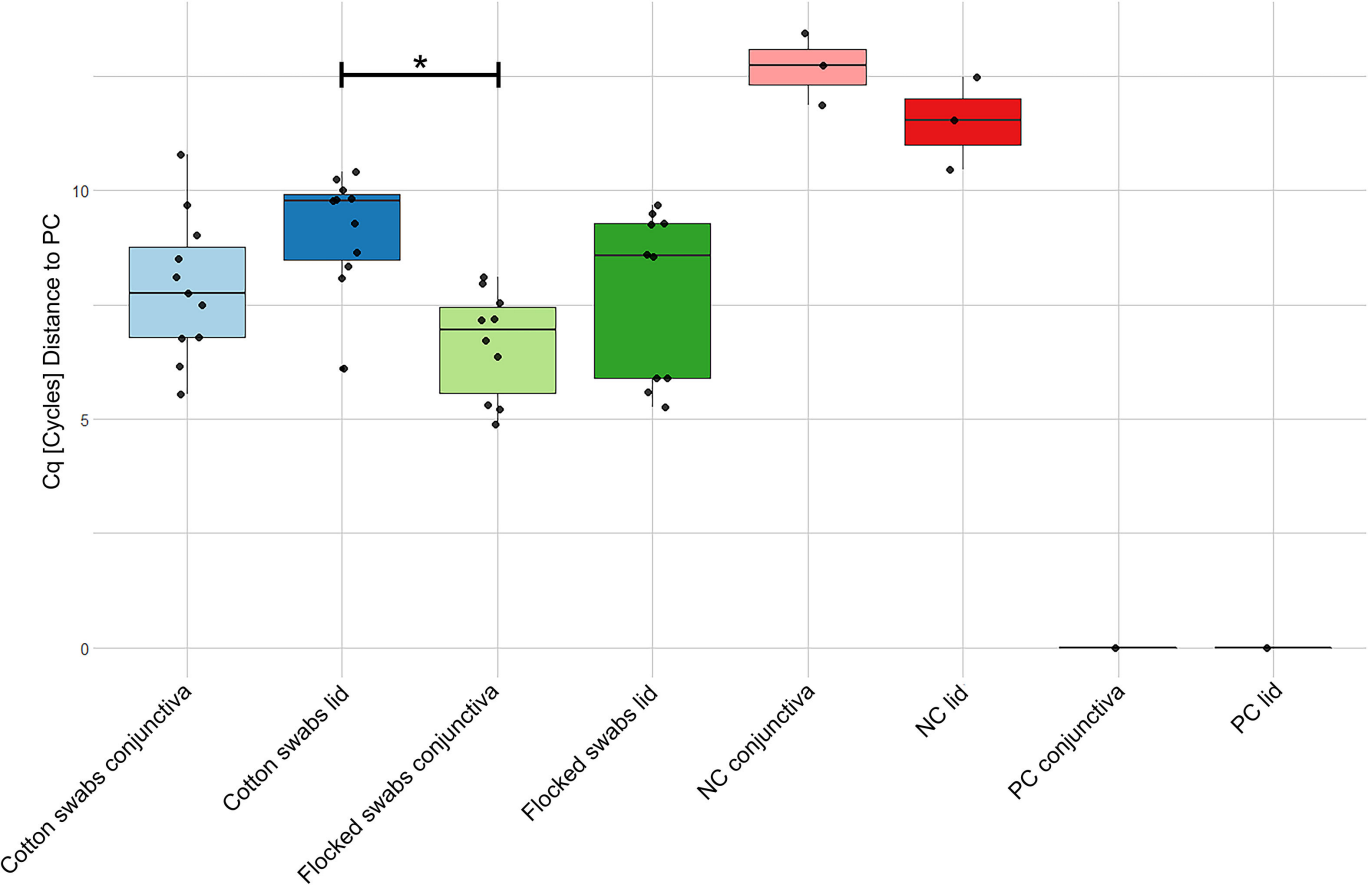
qPCR data of quantification of 16S rRNA genes in samples extracted with the Omega kit. The data was normalized to a positive stool sample control showing stable results over all plates. Note that a lower Cq-value corresponds to a higher 16S rRNA concentration. Cq, Quantification cycle; NC, No-template control; PC, Positive control. * = p-value < 0.05.

qPCR of 16S rRNA genes was performed for lid and conjunctiva samples from both DNA extraction kits (**Figure 3**). Samples extracted with the Qiagen kit could not be distinguished from NTCs and were therefore omitted from the analysis. All Omega samples (except lid samples using cotton swabs) differed from NTCs in their Cq-values. A difference between flocked conjunctiva and cotton lid swabs was found (One-Way ANOVA, p-value = 0.006).

### Microbial reads

A total number of 7’896’779’328 paired-end reads (150 bp) were generated with an average of 74’497’918 (SD = 23’962’936) reads per sample. After trimming of adapter sequences and removal of human reads, 2’842’874 (SD = 6’554’871) high-quality microbial reads per sample remained for subsequent analysis. The Qiagen kit showed an increased ratio of non-human reads divided by the generated sequencing depth (mean Qiagen = 4’308’786 reads/79’023’776 reads = 0.055, mean Omega = 609’102 reads/67’601’372 reads = 0.009, two-tailed *t*-test p = 0.0013). Due to failure of library preparation, the NTCs were not sequenced.

### Taxonomic characterization of the ocular surface microbiome

**Figure 4:**
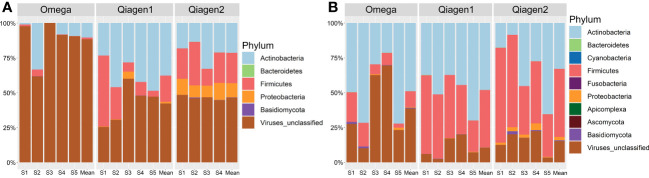
Taxonomic composition at phylum level. The average compositions for each study subject plus the overall mean of the subjects per DNA extraction kit in **(A)** conjunctiva samples and **(B)** lid samples were shown.

In total, 269 microbial species were detected using MetaPhlan3. In both kits, the most abundant bacterial phyla were *Actinobacteria* (29.78% (SD = 28.71) in Omega, 36.62% (SD = 20.50) in Qiagen), *Firmicutes* (6.06% (SD = 8.99) in Omega, 31.22% (SD = 21.03) in Qiagen) and *Proteobacteria* (0.21% (SD = 0.74) in Omega, 3.31% (SD = 5.43) in Qiagen). The relative abundance of viruses differed between the two DNA extraction kits (63.63% (SD = 35.22) in Omega, 28.62% (SD = 21.46) in Qiagen, two-tailed *t*-test p = 3.56E-7) (**Figure 4**). Host DNA contamination in the Omega kit (no host DNA depletion included) averaged at 99.19% (SD = 0.31). In the two Qiagen kits, host DNA contaminations were 98.00% (SD = 1.95) and 92.38% (SD = 9.89), for the Qiagen1 or Qiagen2 batch, respectively.

**Figure 5:**
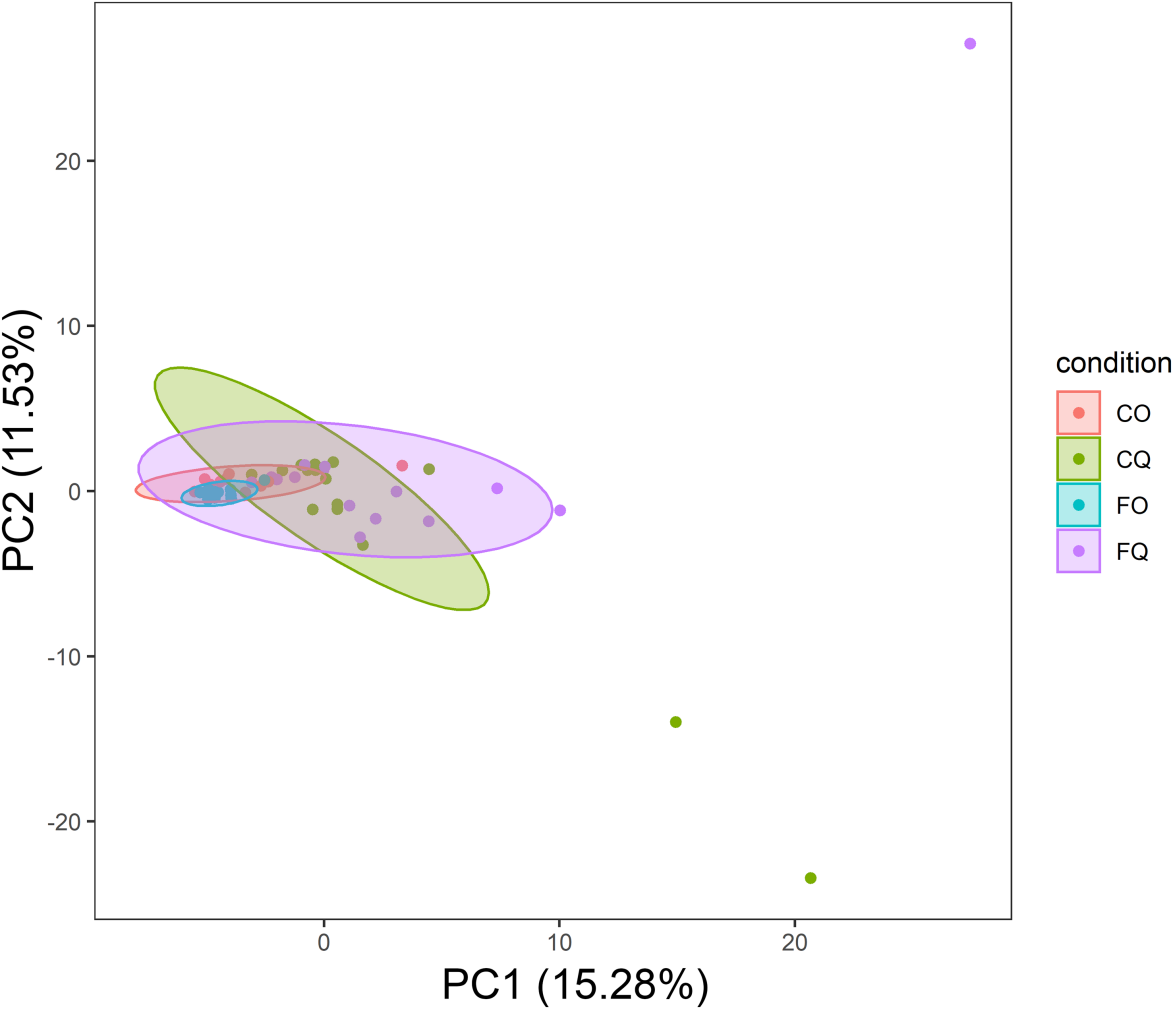
PCA of taxonomic composition in conjunctiva samples. The centroids of the ellipses (95% confidence interval for a multivariate t-distribution) clustered according to the DNA extraction kit. CO, Cotton swabs Omega; CQ, Cotton swabs Qiagen; FO, Flocked swabs Omega; FQ, Flocked swabs Qiagen.

**Figure 6:**
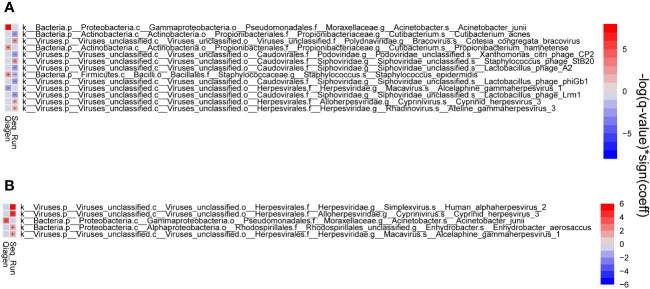
Heat map showing significant associations between metadata and microbial species. Correlations of conjunctiva **(A)** and lid **(B)** samples. Associations in taxonomic composition were found between DNA extraction kits and sequencing run.

The composition of the OSM was dependent on the DNA extraction kit, whereas no differences were found dependent on swab type (**Figures 5**, **6**). Due to the absence of differences from swab type, samples using both types of swabs were analyzed together.

### Comparison of DNA extraction kits

**Figure 7:**
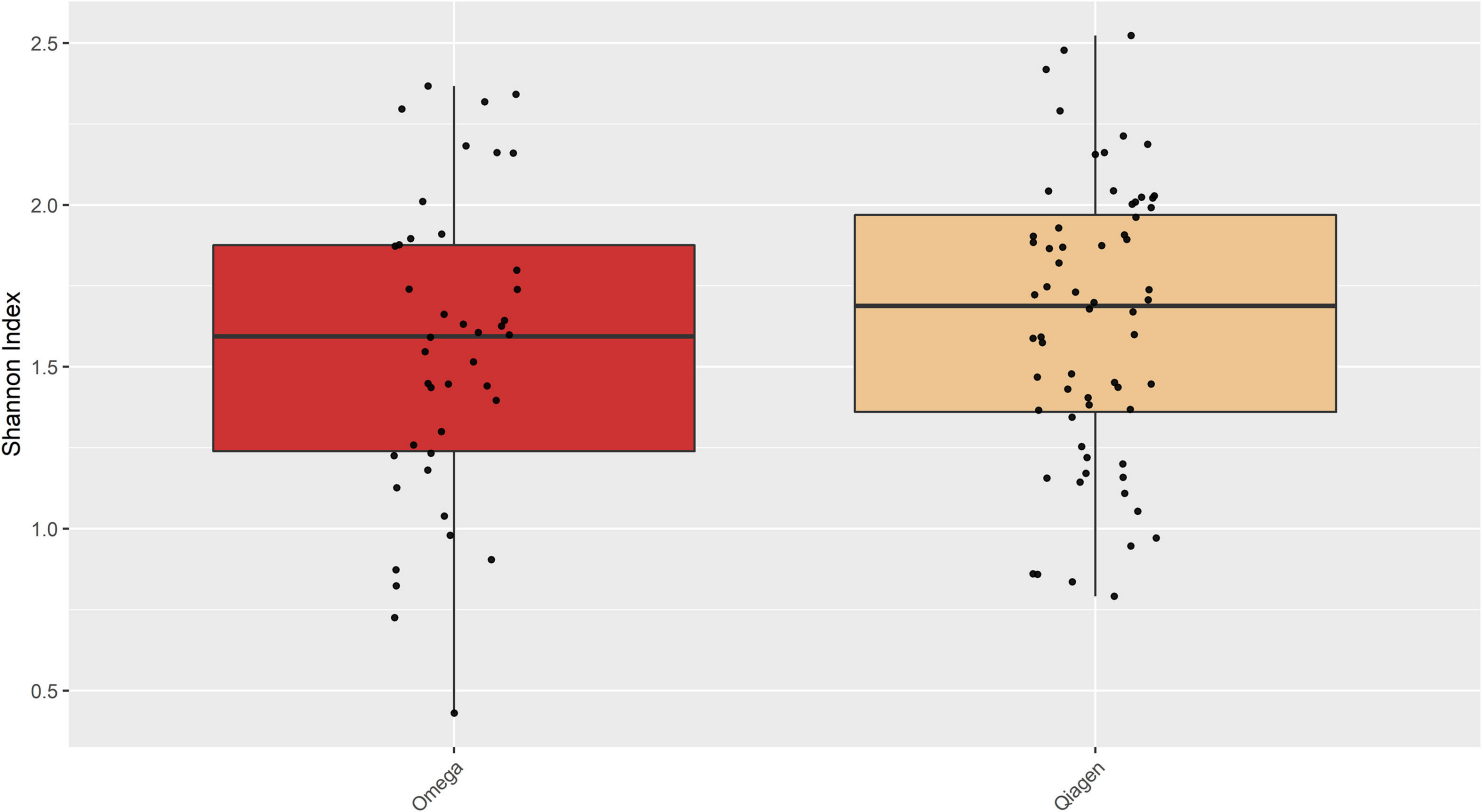
Shannon diversity of the samples extracted with either Omega or Qiagen kit. There was no difference in Shannon diversity observed between the two kits (Student’s *t*-test, p = 0.3664).

OSM samples did not differ in overall species richness (assessed with the Shannon diversity index) depending on the DNA extraction kit (**Figure 7**). A principal component analysis (PCA) showed differences in taxonomic composition in conjunctiva samples according to the extraction kit (Omega or Qiagen, using either cotton or flocked nylon swabs) (**Figure 5**). The same analysis for lid swabs can be found in the supplementary data (**Figure S1**). The taxonomic composition differed between all samples (**Figure 5** and **Figure S1**) that have not been extracted with the same kit in. PERMANOVAs with 1000 permutations were performed on conjunctiva samples (p-values: CO vs CQ = 0.001, CO vs FQ = 0.002, CQ vs FO = 0.001, FO vs FQ = 0.001) and lid samples (p-values: CO vs CQ = 0.001, CO vs FQ = 0.001, CQ vs FO = 0.001, FO vs FQ = 0.001). Samples using different swab types but the same DNA extraction kit did not differ in the taxonomic composition, except for CO vs FO in conjunctiva samples (PERMANOVA, 1000 permutations, p-value = 0.022).

**Figure 8:**
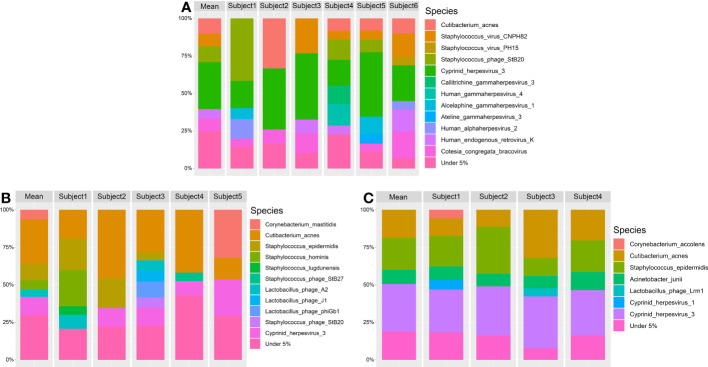
Relative taxonomic composition at species level at over 5% abundance. The mean microbial abundances are shown for each subject. Additionally the mean abundances of all subjects are shown as mean. The DNA extraction kits used are **(A)** Omega, **(B)** Qiagen1 and **(C)** Qiagen2. Taxonomic annotation was performed using MetaPhlan3.

Lid and conjunctiva samples extracted with the Omega kit were dominated by the microbial kingdom viruses (63.63% (SD = 35.22)). The most dominant phyla were viruses (63.63% (SD = 35.22)), *Actinobacteria* (29.78% (SD = 28.71)), *Firmicutes* (6.04% (SD = 8.99)) and *Basidiomycota* (0.32% (SD = 0.67)). The five most abundant species were *Cyprinid herpesvirus 3* (22.90% (SD = 19.78)), *Cutibacterium acnes* (21.29% (SD = 23.42)), *Staphylococcus phage StB20* (6.95% (SD = 13.73)), *Cotesia congregata bracovirus* (5.14% (SD = 7.62)) and *Staphylococcus virus CNPH82* (5.02% (SD = 11.25)) (**Figure 8A**).

In Qiagen samples, bacteria were the predominant microbial kingdom in lid and conjunctiva samples (71.17% (SD = 21.43)), dominated by the phyla *Actinobacteria* (36.62% (SD = 20.50)), *Firmicutes* (31.22% (SD = 21.03)), viruses (28.62% (SD = 21.46)) and *Proteobacteria* (3.31% (SD = 5.43)). The five most abundant species were *Staphylococcus epidermidis* (27.69% (SD = 20.14)), *Cutibacterium acnes* (26.27% (SD = 17.69)), *Cyprinid herpesvirus 3* (13.76% (SD = 13.57)), *Corynebacterium mastitidis* (6.93% (SD = 17.17)) and *Acinetobacter junii* (2.71% (SD = 5.08)) (**Figures 8B, C**). A more detailed graphical representation of the relative abundances of each single measurement in lid and conjunctiva samples can be found in **Figures S2** and **S3**.

### Positive controls

**Figure 9:**
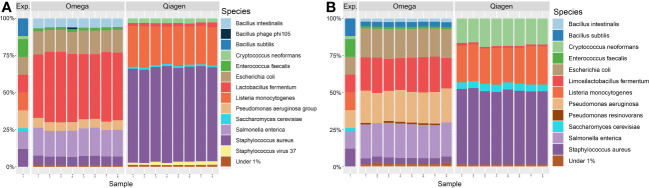
Taxonomic profile of positive controls at species level. Taxonomic annotation of a standardized positive control (ZymoBiomics, Microbial Community Standard D6300) was performed using either **(A)** MetaPhlan3 or **(B)** Kraken2. All 16 samples are technical replicates from the same stock solution. Viruses were detected in extractions from either kit, fungal DNA was only found in Qiagen extractions above the threshold of 1% relative abundance. Exp. = Expected composition.

In order to investigate systematic biases of the DNA extraction kits, technical replicates of a positive control with known microbial composition were processed. All ten expected microbial species were found in extractions of both DNA extraction kits. However, the relative abundances differed from the expected values by up to 51.67% in Qiagen extractions versus 33.39% in Omega extractions. Omega extractions underestimated the presence of *Listeria monocytogenes, Bacillus subtilis* and the two fungal species *Saccharomyces cerevisiae* and *Cryptococcus neoformans*, while overestimating the abundance of *Lactobacillus fermentum*. Extractions with the Qiagen kit underestimated *Bacillus subtilis*, *Enterococcus feacalis*, *Salmonella enterica*, *Escherichia coli* and *Pseudomonas aeruginosa*, while overestimating *Staphylococcus aureus* and *Listeria monocytogenes*. Both fungal species were present at a representative level (**Figure 9A**). In a second approach, the same sequencing reads were analyzed with Kraken2 instead of MetaPhlan3, which also resulted in the detection of all microbial species in the positive control in both kits, but relative abundances differed (**Figure 9B**).

**Figure 10:**
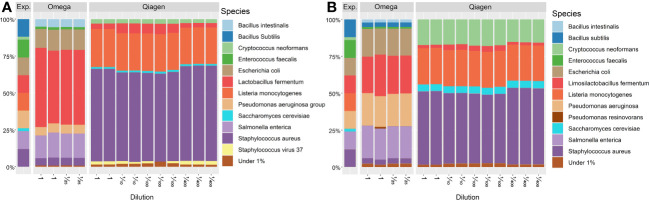
Taxonomic profile of positive controls at species level. Taxonomic annotation of the standardized positive control (ZymoBiomics, Microbial Community Standard D6300) was performed using **(A)** MetaPhlan3 or **(B)** Kraken2. All samples derived from the same stock solution also used in **Figure 9**. -Microbial abundances above a threshold of 1% relative abundance are shown. Exp. = Expected composition. DNA concentrations of Omega dilutions: 1 = 18.75 ng/μl (SD = 0.15), 1/25 = 7.00 ng/μl (SD = 0.04). DNA concentrations of Qiagen dilutions: 1 = 3.46 ng/μl (SD = 0.00), 1/10 = 0.31 ng/μl (SD = 0.01), 1/100 = 0.035 ng/μl (SD = 0.006),1/400 = 0.011 ng/μl (SD = 0.001).

The stability of the sequencing pipeline at low DNA concentration was assessed by dilution of positive controls to the DNA concentration measured in lid and conjunctiva samples. The relative microbial composition of these positive controls was not affected by dilution (**Figure 10**).

## Discussion

With the introduction of modern sequencing technologies, the OSM has been described in much more detail compared to traditional culture techniques. However, due to the low microbial abundance, the characterization of the OSM leads to many challenges. Unlike intestinal microbiome samples, OSM samples are much more prone to contamination introduced in the course of sample collection to data analysis. This is a consequence of low abundant microbes and the resulting high host DNA contaminations. These contaminations may arise from unsterile sampling material, improper techniques or the reagents used for DNA extraction and/or library preparation (Laurence et al., 2014; Salter et al., 2014; Ozkan et al., 2017). In order to account for potential contamination, the inclusion of negative and positive controls for each step of the pipeline is essential. The sequencing depth was set at 60 million reads per sample in order to counteract the filtering of high numbers of host DNA reads. This leaves more microbial reads for analysis, increasing statistical power and decreasing the amount of undetected species (Pereira-Marques et al., 2019), but raising sequencing costs. Other sources of technical bias in the characterization of the OSM such as swabbing pressure (Dong et al., 2011), as well as sources of variability such as age (Zhou et al., 2014; Cavuoto et al., 2018b; Cavuoto et al., 2018a), contact lens wearing (Green et al., 2008; Shin et al., 2016; Stapleton et al., 2017), ocular surface diseases such as blepharitis (Lee et al., 2012), Meibomian gland dysfunction (Watters et al., 2017) or keratitis (Tuzhikov et al., 2013; Song et al., 2015) and the sample source (Ozkan et al., 2018) have been discussed. In this project, we focused on the effect of swab type, sampling location, different DNA extraction kits and taxonomic profiling tools on the taxonomic profile of the OSM using whole-metagenome shotgun sequencing.

The extracted amount of DNA differed between lid and conjunctiva samples, as well as depending on the swab type with flocked nylon swabs collecting more microbes than cotton swabs as previously described (Wise et al., 2021). It is worth noting that, especially for conjunctiva swabs under local anesthesia, all study subjects preferred the cotton swabs for sample collection due to its softer head and less irritation of the conjunctiva. Since less material could be isolated from lid samples, the conjunctiva is the preferred location for ocular surface swabbing, especially in low abundant microbiomes such as the OSM.

The results in **Figure 3** show that bacterial quantification is possible if the DNA concentration is above a certain threshold. In samples extracted with the Qiagen kit, the DNA concentration was below this threshold (**Figure 2**). Another method to estimate the absolute abundance of microbes in the sample is the use of internal standards (ISDs, spike-in controls) (Harrison et al., 2021). Bacterial quantification is important in association studies between the OSM and inflammatory ocular diseases since the bacterial load may be associated with disease development (Graham et al., 2007). Interpretation of 16S rRNA gene qPCR results have to be done in a cautious manner, as the copy number of the 16S rRNA gene is not constant in bacterial genomes ranging from one up to fifteen copies (Vetrovsky and Baldrian, 2013). Thus, a qPCR signal is dependent on microbial community composition.

The microbial compositions of conjunctiva and lid swabs were assessed by whole-metagenome shotgun sequencing, allowing the detection of viral and eukaryotic species in addition to bacterial species. Both fungal and viral communities have been shown to be part of the ocular surface and may contribute to the health of the underlying tissue (Doan et al., 2016; Wen et al., 2017; Shivaji et al., 2019; Shivaji, 2022), making whole-metagenome shotgun sequencing the preferred sequencing choice for the OSM.

Consistent with other studies, the main bacterial phyla present on the OSM are *Actinobacteria*, *Firmicutes* and *Proteobacteria*, while *Bacteroidetes* seems to be more prevalent in studies employing 16S rRNA gene sequencing (Zhou et al., 2014; Doan et al., 2016; Huang et al., 2016; Ozkan et al., 2017; Ham et al., 2018; Dong et al., 2019; Li S. et al., 2019; Li Z. et al., 2019; Yau et al., 2019; Andersson et al., 2021; Kang et al., 2021; Liang et al., 2021; Zhang et al., 2021; Zysset-Burri et al., 2021; Fu et al., 2022). The observed overlap of the main constituents of the OSM reinforce the validity of the presented method to characterize the OSM. Shannon diversity did not differ between the two DNA extraction kits. This result is consistent with a previous study showing no differences in Shannon diversity depending on the extraction method in four out of five different DNA extraction kits (Wagner Mackenzie et al., 2015). While the overall observed structure does not change, the DNA extraction method introduces bias into the relative abundances, impeding cross comparison between studies not employing the same protocols.

In a recently published paper by Delbeke et al., 2023, the authors could not generate sequencing libraries for 16S rRNA sequencing when using DNA extraction kits containing a host DNA depletion step (Delbeke et al., 2023). Our data suggests that the OSM can be characterized without the use of 16S rRNA sequencing even on samples where host DNA has been removed during DNA extraction, reinforcing the use of whole-metagenome shotgun sequencing in OSM research. While whole-metagenome shotgun sequencing does not involve a specific amplification of target DNA, amplification bias could not be eliminated in our approach since input microbial DNA was low concentrated (especially if using the Qiagen extraction kit for DNA isolation) and thus libraries had to be amplified during library preparation. Jones et al., 2015 showed that PCR cycles used for library amplification may lead to a taxonomic bias in bacterial mock communities (Jones et al., 2015). A recent study on the intestinal virome showed that this effect could only be observed by investigating rare viruses (Hsieh et al., 2021). This effect on rare species may be more pronounced in an environment with low microbial abundance such as the OSM.

There is an increased relative abundance of viral DNA in conjunctiva compared to lid swabs, regardless of the DNA extraction kit (**Figure 4**). Although this finding is consistent with previous studies from our lab, the relative abundance of viral DNA is increased in both locations in the current data (Zysset-Burri et al., 2021). This general increase in viral reads may be due to the upgrade from MetaPhlan 2 to MetaPhlan 3 since the database of MetaPhlan 3 contains more than twice as many species than the database of MetaPhlan2 (Truong et al., 2015; Beghini et al., 2021). Furthermore, previous studies showed that different taxonomic profiling tools do not result in equal relative abundances (McIntyre et al., 2017). There are differences in taxonomic output depending on the underlying database as well as on the used algorithm (marker-based versus k-mer-based approach) (Miossec et al., 2020). Since low-abundant species are identified less accurately in marker-based approaches (Miossec et al., 2020), the more resource-intensive k-mer profilers may be appropriate. An interesting tool was presented by Metwally et al., 2016, combining different taxonomic identification methods by weighted voting. This approaches resulted in more accurate results than the individual tools alone (Metwally et al., 2016). Additionally, it cannot be determined if viral DNA originates from proviruses and/or entire viruses. Since the viral to bacterial ratio varied depending on the DNA extraction kit (**Figure 4**), we suppose that, at least not all detected viruses are proviruses.

By comparing ocular surface swabs from the same subjects, we showed that the DNA extraction kit had an effect on the microbial composition, while different swab types did not change the composition (**Figures 5** and **6**). This kit effect may result from the enzymatic host DNA depletion and mechanical lysis via ceramic bead beating steps included in the Qiagen kit. Both steps have been shown to influence the relative abundances of the microbial community (Nandakumar and Marten, 2002; Costea et al., 2017).

In accordance with previous studies, a bias against gram-negative bacteria in positive controls (**Figure 9**) as well as a decrease in the total amount of extracted DNA (**Figure 2**) were observed if microbial DNA was isolated with the Qiagen kit (Horz et al., 2008). This bias originates most likely from the host DNA depletion step since the three gram-negative bacteria in the positive control *Salmonella enterica*, *Escherichia coli* and *Pseudomonas aeruginosa* are underrepresented. This may be due to the mechanism of enzymatic host DNA depletion. To exclude host DNA, host cells are enzymatically lysed while bacterial cells remain intact with subsequent destruction of solved host DNA. Gram-negative bacteria may be more susceptible to this host cell lysis and thus, do not stay intact during treatment. Another explanation may be the partial lysis of susceptible bacteria in the storage solution before host DNA depletion. The two fungal species in the positive control were only found in samples extracted by the Qiagen kit. This may be due to the combination of mechanical and chemical lysis applied in the Qiagen kit, a treatment combination which was shown to increase the detection of fungal species (Janowski et al., 2019). An increased variance in microbial relative abundance due to low DNA concentration could be ruled out by diluting positive controls to the concentration of the OSM samples (**Figure 10**).

Another bias in OSM characterization are errors during microbial annotation. Potential misidentification of microbial species can be observed in our data (**Figure 9**, see *Bacillus intestinalis* and *Bacillus subtilis*). Another case of potential taxonomic misidentification can be seen in **Figure 8**. Even if a high prevalence of herpesviridae DNA on the ocular surface is possible in our cohort, it is unlikely that it originates from the *Cyprinid herpes virus 1* or *3* of the carp family. These misidentifications may be an artefact due to the database-dependent matching of marker sequences, in our case specific for MetaPhlan 3. Reads that do not exactly match all markers of a given species are assigned to the species with the highest overlap. Depending on the database and sequencing method, error rates can reach up to 17% (Edgar, 2018). This may be due to taxonomic mismatching or errors in the curation of the database (Lydon and Lipp, 2018). Data generated by whole-metagenome shotgun sequencing is generally less prone to annotation errors in lower taxonomic ranks compared to 16S rRNA sequencing (Escobar-Zepeda et al., 2018), making it the preferred tool for research where species identification is important.

Limitations of the study include the small sample size and the lack of longitudinal data to assess the temporal stability of the OSM. However, since there is a consistent microbial distribution in all study subjects (**Figure 4**), we suppose that the OSM is stable over the course of sampling in this cohort. Further, the use of anesthetic eye drops, such as tetracaine or oxybuprocaine, is known to inhibit bacterial growth (Labetoulle et al., 2002). However, a more recent study by Delbeke et al., 2022 could not detect a change in overall sequencing results of the OSM after the application of a topical anesthetic (Delbeke et al., 2022).

## Conclusions

This study highlights challenges in the characterization of low abundant microbiomes including the sampling procedure, the selection of the DNA extraction method and the taxonomic profiling tool. Additionally, essential practices such as the inclusion of NTCs and internal standards were investigated for OSM samples which are prone to host and environmental contaminations. Since pipeline optimization is a continuous process and there is no single pipeline that fits all low abundance microbiomes, additional biases will be discovered and have to be accounted for in future projects. Thus, although certain biases during sampling, DNA extraction and sequencing cannot be avoided, careful planning of the pipeline for further research including low abundant microbiomes is crucial.

## Data availability statement

The datasets presented in this study can be found in online repositories. The names of the repository/repositories and accession number(s) can be found below: EMBL's European Bioinformatics Institute (EMBL-EBI), accession PRJEB55147.

## Ethics statement

The studies involving humans were approved by ethics committee of the Canton of Bern (ClinicalTrials.gov: NCT04658238). The studies were conducted in accordance with the local legislation and institutional requirements. The participants provided their written informed consent to participate in this study.

## Author contributions

EH, MZ, and DZ-B contributed to design and conception of the study. MK performed the matching of sequencing data and offered support during statistical analysis. EH wrote the first draft of the manuscript. All authors contributed to manuscript revision and approved the submitted version.

## Supplementary material

Supplementary Figure 1 PCA of taxonomic composition in lid samples. Samples extracted with either the Omega or Qiagen kit differed in their taxonomic composition (p = 0.0010). In all comparisons the employed swab type did not make a difference. CO = Cotton swabs Omega, CQ = Cotton swabs Qiagen, FO = Flocked swabs Omega, FQ = Flocked swabs Qiagen.

Supplementary Figure 2 Relative taxonomic composition of conjunctiva swabs at species level. Samples were extracted with either **(A)** Omega, **(B)** Qiagen1 or **(C)** Qiagen2 DNA extraction kit. Subject samplings on the x axis have 2 to 3 days buffer between them. Taxonomic annotation was performed using MetaPhlan3.

Supplementary Figure 3 Relative taxonomic composition of lid swabs at species level. Samples were extracted with either **(A)** Omega, **(B)** Qiagen1 or **(C)** Qiagen2 DNA extraction kit. Subject samplings on the x axis have 2 to 3 days buffer between them. Taxonomic annotation was performed using MetaPhlan3.

## References

[B1] Andersson J. Vogt J. K. Dalgaard M. D. Pedersen O. Holmgaard K. Heegaard S. (2021). Ocular surface microbiota in patients with aqueous tear-deficient dry eye. Ocul. Surf. 19, 210–217. doi: 10.1016/j.jtos.2020.09.003 32931939

[B2] Andrews S. (2010). FastQC: a quality control tool for high throughput sequence data (Babraham Institute, Cambridge, United Kingdom: Babraham Bioinformatics).

[B3] Beghini F. McIver L. J. Blanco-Miguez A. Dubois L. Asnicar F. Maharjan S.. (2021). Integrating taxonomic, functional, and strain-level profiling of diverse microbial communities with bioBakery 3. Elife 10, e65088. doi: 10.7554/eLife.65088.sa2 33944776 PMC8096432

[B4] Bolger A. M. Lohse M. Usadel B. (2014). Trimmomatic: a flexible trimmer for Illumina sequence data. Bioinformatics 30 (15), 2114–2120. doi: 10.1093/bioinformatics/btu170 24695404 PMC4103590

[B5] Cavuoto K. M. Banerjee S. Miller D. Galor A. (2018a). Composition and comparison of the ocular surface microbiome in infants and older children. Transl. Vis. Sci. Technol. 7 (6), 16. doi: 10.1167/tvst.7.6.16 PMC6269136 30519501

[B6] Cavuoto K. M. Mendez R. Miller D. Galor A. Banerjee S. (2018b). Effect of clinical parameters on the ocular surface microbiome in children and adults. Clin. Ophthalmol. 12, 1189–1197. doi: 10.2147/OPTH.S166547 30013312 PMC6040630

[B7] Costea P. I. Zeller G. Sunagawa S. Pelletier E. Alberti A. Levenez F.. (2017). Towards standards for human fecal sample processing in metagenomic studies. Nat. Biotechnol. 35 (11), 1069–1076. doi: 10.1038/nbt.3960 28967887

[B8] Danecek P. Bonfield J. K. Liddle J. Marshall J. Ohan V. Pollard M. O.. (2021). Twelve years of SAMtools and BCFtools. Gigascience 10 (2), 1–4. doi: 10.1093/gigascience/giab008 PMC7931819 33590861

[B9] Delbeke H. Casteels I. Joossens M. (2022). The effect of topical anesthetics on 16S ribosomal ribonucleic acid amplicon sequencing results in ocular surface microbiome research. Transl. Vis. Sci. Technol. 11 (3), 2. doi: 10.1167/tvst.11.3.2 PMC8899854 35238917

[B10] Delbeke H. Casteels I. Joossens M. (2023). DNA extraction protocol impacts ocular surface microbiome profile. Front. Microbiol. 14, 1128917. doi: 10.3389/fmicb.2023.1128917 37152736 PMC10157640

[B11] Deng Y. Ge X. Li Y. Zou B. Wen X. Chen W.. (2021). Identification of an intraocular microbiota. Cell Discovery 7 (1), 13. doi: 10.1038/s41421-021-00245-6 33750767 PMC7943566

[B12] Doan T. Akileswaran L. Andersen D. Johnson B. Ko N. Shrestha A.. (2016). Paucibacterial microbiome and resident DNA virome of the healthy conjunctiva. Invest. Ophthalmol. Visual Sci. 57 (13), 5116–5126. doi: 10.1167/iovs.16-19803 27699405 PMC5054734

[B13] Dong Q. Brulc J. M. Iovieno A. Bates B. Garoutte A. Miller D.. (2011). Diversity of bacteria at healthy human conjunctiva. Invest. Ophthalmol. Visual Sci. 52 (8), 5408–5413. doi: 10.1167/iovs.10-6939 21571682 PMC3176057

[B14] Dong X. Wang Y. Wang W. Lin P. Huang Y. (2019). Composition and diversity of bacterial community on the ocular surface of patients with meibomian gland dysfunction. Invest. Ophthalmol. Visual Sci. 60 (14), 4774–4783. doi: 10.1167/iovs.19-27719 31738825

[B15] Edgar R. (2018). Taxonomy annotation and guide tree errors in 16S rRNA databases. PeerJ 6, e5030. doi: 10.7717/peerj.5030 29910992 PMC6003391

[B16] Escobar-Zepeda A. Godoy-Lozano E. E. Raggi L. Segovia L. Merino E. Gutierrez-Rios R. M.. (2018). Analysis of sequencing strategies and tools for taxonomic annotation: Defining standards for progressive metagenomics. Sci. Rep. 8 (1), 12034. doi: 10.1038/s41598-018-30515-5 30104688 PMC6089906

[B17] Fu Y. Wu J. Wang D. Li T. Shi X. Li L.. (2022). Metagenomic profiling of ocular surface microbiome changes in Demodex blepharitis patients. Front. Cell. Infect. Microbiol. 12, 922753. doi: 10.3389/fcimb.2022.922753 35937693 PMC9354880

[B18] Galazzo G. van Best N. Benedikter B. J. Janssen K. Bervoets L. Driessen C.. (2020). How to count our microbes? The effect of different quantitative microbiome profiling approaches. Front. Cell. Infect. Microbiol. 10, 403. doi: 10.3389/fcimb.2020.00403 32850498 PMC7426659

[B19] Ganda E. Beck K. L. Haiminen N. Silverman J. D. Kawas B. Cronk B. D.. (2021). DNA extraction and host depletion methods significantly impact and potentially bias bacterial detection in a biological fluid. mSystems 6 (3), e00619–e00621. doi: 10.1128/mSystems.00619-21 34128697 PMC8574158

[B20] Glassing A. Dowd S. E. Galandiuk S. Davis B. Chiodini R. J. (2016). Inherent bacterial DNA contamination of extraction and sequencing reagents may affect interpretation of microbiota in low bacterial biomass samples. Gut. Pathog. 8, 24. doi: 10.1186/s13099-016-0103-7 27239228 PMC4882852

[B21] Graham J. E. Moore J. E. Jiru X. Moore J. E. Goodall E. A. Dooley J. S.. (2007). Ocular pathogen or commensal: a PCR-based study of surface bacterial flora in normal and dry eyes. Invest. Ophthalmol. Visual Sci. 48 (12), 5616–5623. doi: 10.1167/iovs.07-0588 18055811

[B22] Green M. Apel A. Stapleton F. (2008). Risk factors and causative organisms in microbial keratitis. Cornea 27 (1), 22–27. doi: 10.1097/ICO.0b013e318156caf2 18245962

[B23] Ham B. Hwang H. B. Jung S. H. Chang S. Kang K. D. Kwon M. J. (2018). Distribution and diversity of ocular microbial communities in diabetic patients compared with healthy subjects. Curr. Eye. Res. 43 (3), 314–324. doi: 10.1080/02713683.2017.1406528 29172724

[B24] Harrison J. G. John Calder W. Shuman B. Alex Buerkle C. (2021). The quest for absolute abundance: The use of internal standards for DNA-based community ecology. Mol. Ecol. Resour. 21 (1), 30–43. doi: 10.1111/1755-0998.13247 32889760

[B25] Heravi F. S. Zakrzewski M. Vickery K. Hu H. (2020). Host DNA depletion efficiency of microbiome DNA enrichment methods in infected tissue samples. J. Microbiol. Methods 170, 105856. doi: 10.1016/j.mimet.2020.105856 32007505

[B26] Horz H. P. Scheer S. Huenger F. Vianna M. E. Conrads G. (2008). Selective isolation of bacterial DNA from human clinical specimens. J. Microbiol. Methods 72 (1), 98–102. doi: 10.1016/j.mimet.2007.10.007 18053601

[B27] Hsieh S. Y. Tariq M. A. Telatin A. Ansorge R. Adriaenssens E. M. Savva G. M.. (2021). Comparison of PCR versus PCR-free DNA library preparation for characterising the human faecal virome. Viruses 13 (10), 1–19. doi: 10.3390/v13102093 PMC8537689 34696523

[B28] Huang Y. Yang B. Li W. (2016). Defining the normal core microbiome of conjunctival microbial communities. Clin. Microbiol. Infect. Off. Publ. Eur. Soc. Clin. Microbiol. Infect. Dis. 22 (7), 643 e7–64 e12. doi: 10.1016/j.cmi.2016.04.008 27102141

[B29] Human Microbiome Project C (2012a). Structure, function and diversity of the healthy human microbiome. Nature 486 (7402), 207–214. doi: 10.1038/nature11234 22699609 PMC3564958

[B30] Human Microbiome Project C (2012b). A framework for human microbiome research. Nature 486 (7402), 215–221. doi: 10.1038/nature11209 22699610 PMC3377744

[B31] Janowski D. Wilgan R. Leski T. Karliński L. Rudawska M. (2019). Effective molecular identification of ectomycorrhizal fungi: revisiting DNA isolation methods. Forests 10 (3), 218. doi: 10.3390/f10030218

[B32] Jones M. B. Highlander S. K. Anderson E. L. Li W. Dayrit M. Klitgord N.. (2015). Library preparation methodology can influence genomic and functional predictions in human microbiome research. Proc. Natl. Acad. Sci. United. States America. 112 (45), 14024–14029. doi: 10.1073/pnas.1519288112 PMC4653211 26512100

[B33] Kang Y. Lin S. Ma X. Che Y. Chen Y. Wan T.. (2021). Strain heterogeneity, cooccurrence network, taxonomic composition and functional profile of the healthy ocular surface microbiome. Eye. Vis. (Lond). 8 (1), 6. doi: 10.1186/s40662-021-00228-4 33622400 PMC7903678

[B34] Keilty R. A. (1930). The bacterial flora of the normal conjunctiva with comparative nasal culture study. Am. J. Ophthalmol. 13 (10), 876–879. doi: 10.1016/S0002-9394(30)92437-3

[B35] Labetoulle M. Frau E. Offret H. Nordmann P. Naas T. (2002). Non-preserved 1% lidocaine solution has less antibacterial properties than currently available anaesthetic eye-drops. Curr. Eye. Res. 25 (2), 91–97. doi: 10.1076/ceyr.25.2.91.10159 12525962

[B36] Langmead B. Salzberg S. L. (2012). Fast gapped-read alignment with Bowtie 2. Nat. Methods 9 (4), 357–359. doi: 10.1038/nmeth.1923 22388286 PMC3322381

[B37] Lauder A. P. Roche A. M. Sherrill-Mix S. Bailey A. Laughlin A. L. Bittinger K.. (2016). Comparison of placenta samples with contamination controls does not provide evidence for a distinct placenta microbiota. Microbiome 4 (1), 29. doi: 10.1186/s40168-016-0172-3 27338728 PMC4917942

[B38] Laurence M. Hatzis C. Brash D. E. (2014). Common contaminants in next-generation sequencing that hinder discovery of low-abundance microbes. PloS One 9 (5), e97876. doi: 10.1371/journal.pone.0097876 24837716 PMC4023998

[B39] Lee S. H. Oh D. H. Jung J. Y. Kim J. C. Jeon C. O. (2012). Comparative ocular microbial communities in humans with and without blepharitis. Invest. Ophthalmol. Visual Sci. 53 (9), 5585–5593. doi: 10.1167/iovs.12-9922 22836761

[B40] Li Z. Gong Y. Chen S. Li S. Zhang Y. Zhong H.. (2019). Comparative portrayal of ocular surface microbe with and without dry eye. J. Microbiol. 57 (11), 1025–1032. doi: 10.1007/s12275-019-9127-2 31463790

[B41] Li S. Yi G. Peng H. Li Z. Chen S. Zhong H.. (2019). How ocular surface microbiota debuts in type 2 diabetes mellitus. Front. Cell. Infect. Microbiol. 9, 202. doi: 10.3389/fcimb.2019.00202 31263683 PMC6590198

[B42] Liang X. Li Y. Xiong K. Chen S. Li Z. Zhang Z.. (2021). Demodex infection changes ocular surface microbial communities, in which meibomian gland dysfunction may play a role. Ophthalmol. Ther. 10 (3), 601–617. doi: 10.1007/s40123-021-00356-z 34159561 PMC8319250

[B43] Lu J. Breitwieser F. P. Thielen P. Salzberg S. L. (2017). Bracken: estimating species abundance in metagenomics data. PeerJ. Comput. Sci. 3, e104. doi: 10.7717/peerj-cs.104 PMC12016282 40271438

[B44] Lydon K. A. Lipp E. K. (2018). Taxonomic annotation errors incorrectly assign the family Pseudoalteromonadaceae to the order Vibrionales in Greengenes: implications for microbial community assessments. PeerJ 6, e5248. doi: 10.7717/peerj.5248 30018864 PMC6044269

[B45] Marotz C. A. Sanders J. G. Zuniga C. Zaramela L. S. Knight R. Zengler K. (2018). Improving saliva shotgun metagenomics by chemical host DNA depletion. Microbiome 6 (1), 42. doi: 10.1186/s40168-018-0426-3 29482639 PMC5827986

[B46] McDermott A. M. (2013). Antimicrobial compounds in tears. Exp. Eye. Res. 117, 53–61. doi: 10.1016/j.exer.2013.07.014 23880529 PMC3844110

[B47] McIntyre A. B. R. Ounit R. Afshinnekoo E. Prill R. J. Henaff E. Alexander N.. (2017). Comprehensive benchmarking and ensemble approaches for metagenomic classifiers. Genome Biol. 18 (1), 182. doi: 10.1186/s13059-017-1299-7 28934964 PMC5609029

[B48] Metwally A. A. Dai Y. Finn P. W. Perkins D. L. (2016). WEVOTE: weighted voting taxonomic identification method of microbial sequences. PloS One 11 (9), e0163527. doi: 10.1371/journal.pone.0163527 27683082 PMC5040256

[B49] Miossec M. J. Valenzuela S. L. Perez-Losada M. Johnson W. E. Crandall K. A. Castro-Nallar E. (2020). Evaluation of computational methods for human microbiome analysis using simulated data. PeerJ 8, e9688. doi: 10.7717/peerj.9688 32864214 PMC7427543

[B50] Nandakumar M. P. Marten M. R. (2002). Comparison of lysis methods and preparation protocols for one- and two-dimensional electrophoresis of Aspergillus oryzae intracellular proteins. Electrophoresis 23 (14), 2216–2222. doi: 10.1002/1522-2683(200207)23:14<2216::AID-ELPS2216>3.0.CO;2-Y 12210225

[B51] Ozkan J. Coroneo M. Willcox M. Wemheuer B. Thomas T. (2018). Identification and visualization of a distinct microbiome in ocular surface conjunctival tissue. Invest. Ophthalmol. Visual Sci. 59 (10), 4268–4276. doi: 10.1167/iovs.18-24651 30140925

[B52] Ozkan J. Nielsen S. Diez-Vives C. Coroneo M. Thomas T. Willcox M. (2017). Temporal stability and composition of the ocular surface microbiome. Sci. Rep. 7 (1), 9880. doi: 10.1038/s41598-017-10494-9 28852195 PMC5575025

[B53] Pereira-Marques J. Hout A. Ferreira R. M. Weber M. Pinto-Ribeiro I. van Doorn L. J.. (2019). Impact of host DNA and sequencing depth on the taxonomic resolution of whole metagenome sequencing for microbiome analysis. Front. Microbiol. 10, 1277. doi: 10.3389/fmicb.2019.01277 31244801 PMC6581681

[B54] Salter S. J. Cox M. J. Turek E. M. Calus S. T. Cookson W. O. Moffatt M. F.. (2014). Reagent and laboratory contamination can critically impact sequence-based microbiome analyses. BMC Biol. 12 (1), 1–12. doi: 10.1186/s12915-014-0087-z 25387460 PMC4228153

[B55] Shin H. Price K. Albert L. Dodick J. Park L. Dominguez-Bello M. G. (2016). Changes in the eye microbiota associated with contact lens wearing. mBio 7 (2), e00198. doi: 10.1128/mBio.00198-16 27006462 PMC4817251

[B56] Shivaji S. (2022). Virome of the healthy human eye. Hum. Ocular. Microbiome.: Springer; p, 225–239. doi: 10.1007/978-981-19-1754-7_8

[B57] Shivaji S. Jayasudha R. Sai Prashanthi G. Kalyana Chakravarthy S. Sharma S. (2019). The human ocular surface fungal microbiome. Invest. Ophthalmol. Visual Sci. 60 (1), 451–459. doi: 10.1167/iovs.18-26076 30703210

[B58] Shovlin J. P. Argüeso P. Carnt N. Chalmers R. L. Efron N. Fleiszig S. M.. (2013). 3. Ocular surface health with contact lens wear. Contact. Lens. Anterior. Eye. 36, S14–S21. doi: 10.1016/S1367-0484(13)60005-3 23347571

[B59] Song H.-Y. Qiu B.-F. Liu C. ZHu S.-X. Wang S.-C. Miao J.. (2015). Identification of causative pathogens in mouse eyes with bacterial keratitis by sequence analysis of 16S rDNA libraries. Exp. Animals. 64 (1), 49–56. doi: 10.1538/expanim.14-0046 PMC4329515 25312507

[B60] Stapleton F. Naduvilath T. Keay L. Radford C. Dart J. Edwards K.. (2017). Risk factors and causative organisms in microbial keratitis in daily disposable contact lens wear. PloS One 12 (8), e0181343. doi: 10.1371/journal.pone.0181343 28813424 PMC5558933

[B61] St Leger A. J. Desai J. V. Drummond R. A. Kugadas A. Almaghrabi F. Silver P.. (2017). An Ocular Commensal Protects against Corneal Infection by Driving an Interleukin-17 Response from Mucosal gammadelta T Cells. Immunity 47 (1), 148–58.e5. doi: 10.1016/j.immuni.2017.06.014 28709803 PMC5553552

[B62] Suzuki T. Sutani T. Nakai H. Shirahige K. Kinoshita S. (2020). The microbiome of the meibum and ocular surface in healthy subjects. Invest. Ophthalmol. Visual Sci. 61 (2), 18. doi: 10.1167/iovs.61.2.18 PMC7326502 32053729

[B63] Truong D. T. Franzosa E. A. Tickle T. L. Scholz M. Weingart G. Pasolli E.. (2015). MetaPhlAn2 for enhanced metagenomic taxonomic profiling. Nat. Methods 12 (10), 902–903. doi: 10.1038/nmeth.3589 26418763

[B64] Tuzhikov A. Dong Q. Panchin A. Thanathanee O. Shalabi N. Nelson D.. (2013). Keratitis-induced changes to the homeostatic microbiome at the human cornea. Invest. Ophthalmol. Visual Sci. 54 (15), 2891.

[B65] Ueta M. Kinoshita S. (2010). Innate immunity of the ocular surface. Brain Res. Bull. 81 (2-3), 219–228. doi: 10.1016/j.brainresbull.2009.10.001 19828129

[B66] Vetrovsky T. Baldrian P. (2013). The variability of the 16S rRNA gene in bacterial genomes and its consequences for bacterial community analyses. PloS One 8 (2), e57923. doi: 10.1371/journal.pone.0057923 23460914 PMC3583900

[B67] Wagner Mackenzie B. Waite D. W. Taylor M. W. (2015). Evaluating variation in human gut microbiota profiles due to DNA extraction method and inter-subject differences. Front. Microbiol. 6, 130. doi: 10.3389/fmicb.2015.00130 25741335 PMC4332372

[B68] Watters G. A. Turnbull P. R. Swift S. Petty A. Craig J. P. (2017). Ocular surface microbiome in meibomian gland dysfunction. Clin. Exp. Ophthalmol. 45 (2), 105–111. doi: 10.1111/ceo.12810 27473509

[B69] Wen X. Miao L. Deng Y. Bible P. W. Hu X. Zou Y.. (2017). The influence of age and sex on ocular surface microbiota in healthy adults. Invest. Ophthalmol. Visual Sci. 58 (14), 6030–6037. doi: 10.1167/iovs.17-22957 29196767

[B70] Wise N. M. Wagner S. J. Worst T. J. Sprague J. E. Oechsle C. M. (2021). Comparison of swab types for collection and analysis of microorganisms. Microbiologyopen 10 (6), e1244. doi: 10.1002/mbo3.1244 34964289 PMC8591448

[B71] Wood D. E. Lu J. Langmead B. (2019). Improved metagenomic analysis with Kraken 2. Genome Biol. 20 (1), 257. doi: 10.1186/s13059-019-1891-0 31779668 PMC6883579

[B72] Yau J. W. Hou J. Tsui S. K. W. Leung T. F. Cheng N. S. Yam J. C.. (2019). Characterization of ocular and nasopharyngeal microbiome in allergic rhinoconjunctivitis. Pediatr. Allergy Immunol. 30 (6), 624–631. doi: 10.1111/pai.13088 31132163

[B73] Zhang Z. Zou X. Xue W. Zhang P. Wang S. Zou H. (2021). Ocular surface microbiota in diabetic patients with dry eye disease. Invest. Ophthalmol. Visual Sci. 62 (12), 13. doi: 10.1167/iovs.62.12.13 PMC8444464 34524384

[B74] Zhou Y. Holland M. J. Makalo P. Joof H. Ch R. Mabey D. C. W.. (2014). The conjunctival microbiome in health and trachomatous disease: a case control study. Genome Med. 6 (11), 99. doi: 10.1186/s13073-014-0099-x 25484919 PMC4256740

[B75] Zysset-Burri D. C. Schlegel I. Lincke J. B. Jaggi D. Keller I. Heller M.. (2021). Understanding the interactions between the ocular surface microbiome and the tear proteome. Invest. Ophthalmol. Visual Sci. 62 (10), 8. doi: 10.1167/iovs.62.10.8 PMC8354087 34369983

