# Supplementary figures and images for: Challenges and insights in the exploration of the low abundance human ocular surface microbiome
Source: Front Cell Infect Microbiol. 2023 Sep 1;13:1232147. doi: 10.3389/fcimb.2023.1232147 (PMC10505673; doi:10.3389/fcimb.2023.1232147)

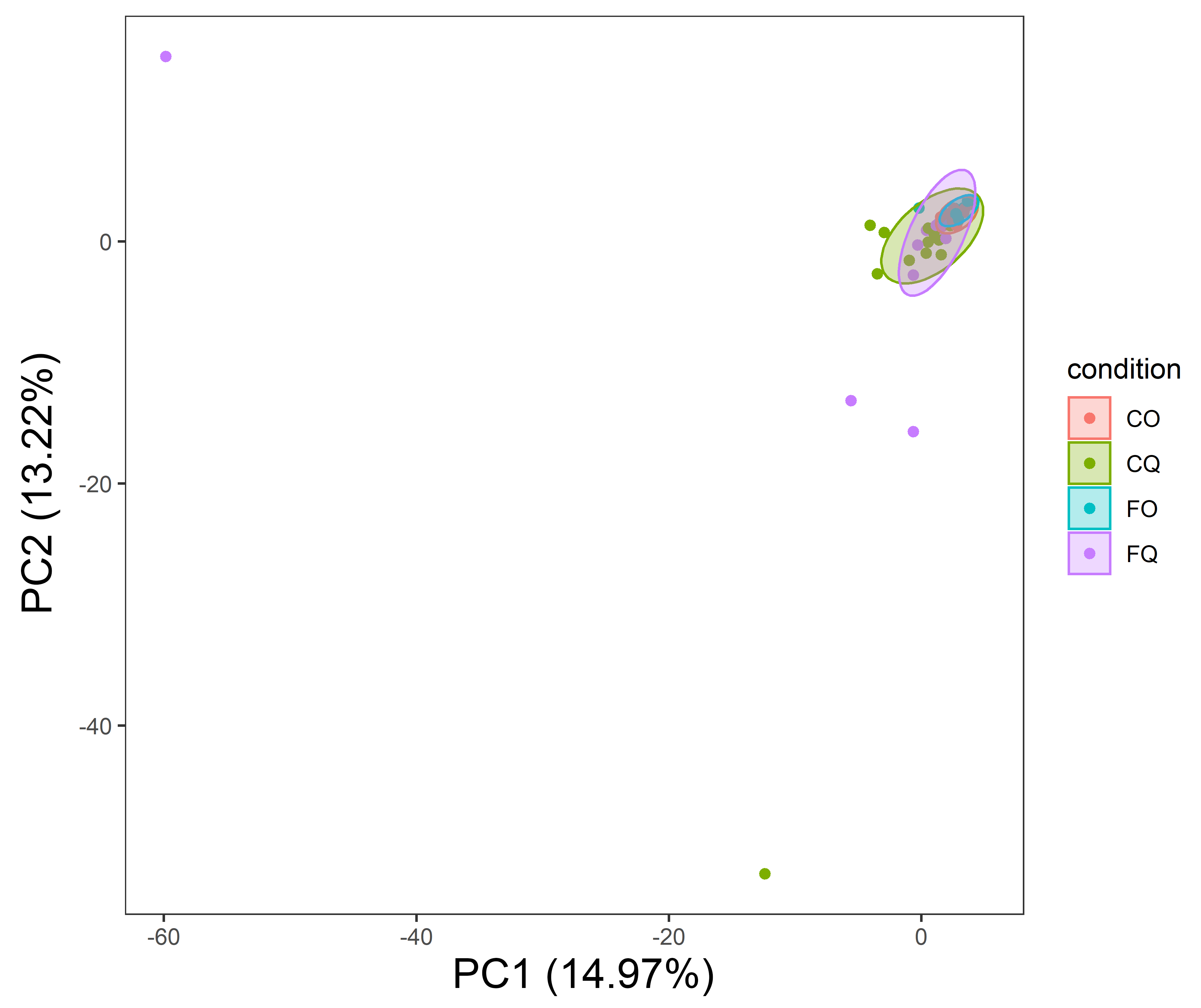

Supplement: Supplementary Figure 1 — PCA of taxonomic composition in lid samples. Samples extracted with either the Omega or Qiagen kit differed in their taxonomic composition (p = 0.0010). In all comparisons the employed swab type did not make a difference. CO = Cotton swabs Omega, CQ = Cotton swabs Qiagen, FO = Flocked swabs Omega, FQ = Flocked swabs Qiagen. [file Image_1.tif]
