# Supplementary material for: Challenges and insights in the exploration of the low abundance human ocular surface microbiome
Source: Front Cell Infect Microbiol. 2023 Sep 1;13:1232147. doi: 10.3389/fcimb.2023.1232147 (PMC10505673; doi:10.3389/fcimb.2023.1232147)
Supplement: Supplementary file 4 [file DataSheet_1.pdf]

Supporting material

S1: Alteration to the provided kit protocol for swab samples

Omega Kit: Before the addition of BL buffer, 2 µl of RNase A (#19101, QIAGEN, Hilden, Germany) were added and incubated for 2 minutes. The optional column equilibration step was omitted.

Qiagen Kit: The protocol of Qiagen was only altered in the first step, where swabs were not only swirled in 1 ml of ice cooled DPBS 1X before the addition of AHL buffer, but vortexed at full speed for one minute. In step six, the pathogen lysis tubes were put into a tissue lyser (Precellys 24, Bertin instruments, Montigny-le-Bretonneux, France) at 6800 rpm equivalent to 5.5 m/s on a FastPrep-24 according to the PowerLyzer 24 user manual. DNA was eluted from the silica columns with 50 µl of either AVE buffer (RNase-free water with 0.04% sodium azide NaN3) for Qiagen samples or 50 µl elution buffer (10 mM Tris-HCl, pH 8.5) for Omega samples.
